# Fatty Acid Metabolism in Shading-Induced Tolerance of Star Ruby Grapefruit to Postharvest Chilling Injury

**DOI:** 10.3390/plants14243848

**Published:** 2025-12-17

**Authors:** Aurora Lozano-Omeñaca, María-Jesús Rodrigo, Lorenzo Zacarías

**Affiliations:** Instituto de Agroquímica y Tecnología de Alimentos (IATA), Consejo Superior de Investigaciones Científicas (CSIC), Catedrático Agustín Escardino 7, 46980 Paterna, Spain; aurora.lozano@iata.csic.es (A.L.-O.); mjrodrigo@iata.csic.es (M.-J.R.)

**Keywords:** chilling injury, fatty acids, desaturase genes expression, grapefruit, cold storage, preharvest factors

## Abstract

Chilling injury (CI) is a major postharvest disorder in citrus fruit, and fatty acid (FA) metabolism has been proposed as a key determinant of cold tolerance. We have investigated this relationship in the fruit of the Star Ruby grapefruit and found that preharvest fruit shading induces lycopene accumulation in the peel and tolerance to CI during subsequent cold storage in comparison with uncovered fruits, which were sensitive to CI. Then, FA profiling and the expression of FA desaturation (*FADs*) genes were examined in the flavedo of covered (C) and non-covered (NC) grapefruits during 8 weeks of storage at 2 °C. Linoleic acid was the predominant unsaturated FA in the flavedo of Star Ruby grapefruit, which accumulated more highly in the CI-sensitive than in the CI-tolerant fruit at harvest and during the whole storage period. Interestingly, C and NC fruit also exhibited distinct *FAD* transcriptional signatures at harvest, suggesting the influence of preharvest factors. Cold storage stimulated FA desaturation in both C and NC fruit and differentially affected the expression of *FADs* genes during cold storage. These results demonstrate that FA metabolism and regulation of *FADs* expression are tightly connected with preharvest factors that may modulate the response of grapefruits to postharvest cold storage.

## 1. Introduction

Chilling injury (CI) is a significant postharvest physiological disorder affecting *Citrus* fruit quality, limiting its commercialization in the fresh fruit market and causing important economic losses and food waste. In particular, due to their subtropical origin, the fruit of many *Citrus* species and varieties may develop CI when subjected to low non-freezing temperatures for prolonged storage periods [1]. CI in *Citrus* fruits is manifested by a myriad of symptoms in the peel, such as pitting, superficial scalding, browning, or, in severe cases, large necrotic and shrunken regions in the flavedo [1,2]. The development of CI symptoms in *Citrus* fruit is species- and genotype-dependent but is highly influenced by numerous environmental and agronomical factors, including rootstock, fruit maturity, and canopy position, among others [2,3]. Therefore, understanding the influence of these variables on the incidence of CI and their metabolic impact is crucial for effective CI prediction and management during postharvest cold storage [2].

Grapefruits (*Citrus paradisi*) are among the *Citrus* species genetically more sensitive to develop CI during postharvest storage [4,5]. The appearance of CI symptoms in grapefruits is characterized by the emergence of small brown pits on the fruit surface that evolve into shrunken brown spots and extended depressed areas on the peel after prolonged cold storage periods [2]. Preharvest factors, such as light incidence during fruit maturation, have been proven to influence peel composition and postharvest CI tolerance in grapefruit [4,6]. Specifically, research in Star Ruby grapefruit has revealed that shielding the fruit from light by bagging during the later stages of development promoted accumulation of lycopene, a red carotenoid with antioxidant activity, in the peel, significantly reducing the incidence of CI during cold storage [7]. Moreover, natural light incidence through canopy position of the fruit has been proven to impact CI tolerance in grapefruit [8] and modified rind quality and composition during postharvest storage in other citrus species [9,10]. Other studies have also documented metabolic changes induced by light deprivation in citrus flavedo during fruit maturation, including carotenoid biosynthesis [4,11,12], tocopherol accumulation [6], and the implication of the antioxidant system in CI susceptibility [7,13].

The prevailing hypothesis regarding the cellular and molecular mechanisms that trigger chilling injury in plants postulates that low-temperature stress induces a phase transition in lipid membranes from a fluid liquid phase to a solid gel state [14]. The structural shift in membranes results in a loss of fluidity and an increase in permeability, contributing to electrolyte leakage and oxidative membrane damage, with both events associated with CI [15,16]. The increase in unsaturated fatty acids (UFA) levels in the composition of the membranes is part of the adaptation mechanism to cold stress since their structural double bonds contribute to the maintenance of membrane fluidity and the prevention of oxidative damage [17,18,19]. The correlation between fatty acid (FA) unsaturation levels and the susceptibility to CI development has been established in various subtropical and tropical fruits, including pineapple [20], loquat [21], kiwifruit [22], guava fruit [23], papaya [24], and banana [25]. Recently, this relationship has also been established in mandarin fruits, where a higher content of UFAs was found in the peel of a CI-sensitive mandarin at harvest compared with that of a CI-tolerant genotype [26]. Consequently, many studies highlight the importance of the genes regulating the synthesis of UFAs, fatty acid desaturases (FAD), and their transcriptomic changes in cold stress response [17]. In higher plants, the most relevant UFAs in membrane lipids are 18-carbon (C18) fatty acids [27]. The enzymes responsible for their synthesis have been described and analyzed in silico for the *Citrus* genome [26]. Briefly, C18 fatty acid synthesis starts at the plastid with the elongation of palmitic acid (C16:0-ACP, acyl-carrier-protein) to stearic acid (C18:0-ACP) by β-ketoacyl-ACP synthases (KASII) and the first double-bond insertion into oleic acid (C18:1-ACP) by stearoyl-ACP desaturases (SADs) [28]. Following this, desaturations for the synthesis of linoleic (C18:2) and linolenic acid (C18:3) can take place in the plastids but mostly occur in the endoplasmic reticulum by oleate desaturases (FAD2 and FAD6) and linoleate desaturases (FAD3 and FAD7/8), respectively [27]. Organelle compartmentalization is a relevant characteristic of the FA desaturation process in higher plants, hence the importance of acyl-ACP thioesterases (FATs) and acyl-CoA synthetases (LACSs) as intermediaries of fatty acid and lipid trafficking [26,29]. The expression of *FAD* genes during cold storage has been linked to CI susceptibility in the fruit of different species, such as olives [30,31], peach [32], apple [33], banana fruit [25], and citrus [26,34,35,36]. However, further research is necessary to elucidate the different factors involved in this relationship in fruits of other *Citrus* species.

In a previous study, we showed a lower UFA content at harvest in a naturally chilling-tolerant genotype of mandarin than in the chilling-sensitive genotype [26]. In order to understand the involvement of FA metabolism in the tolerance of *Citrus* fruits to CI in more detail, we took advantage of Star Ruby grapefruits’ tolerance to chilling induced by fruit shading during the last stages of development [6,7]. Shading increased lycopene levels in grapefruit flavedo, potentially reducing membrane lipid oxidation [7,13] and helping to maintain membrane integrity alongside FA desaturation, although the molecular interplay between these pathways is still unclear. Interestingly, the influence of preharvest light exposure on the composition of FA in fruits has been reported in several systems, such as tomato [37], olive drupes [38,39], and cucumber [40], and changes have been observed in the transcriptional regulation of *FAD* genes in walnut [41], olive drupes [42], cotton [43], and soybean [44]. We hypothesized that the tolerance to postharvest CI, which was induced by shading, in the fruit of Star Ruby grapefruits may be modulated by changes in FA metabolism. Based on this, the objective of this work was to analyze FA profiling and the expression of desaturation-related genes in the flavedo of shaded (tolerant to CI) and non-shaded (sensitive to CI) Star Ruby grapefruits at harvest and during storage at 2 °C for up to 8 weeks.

## 2. Results

### 2.1. Chilling Injury of Covered and Non-Covered Star Ruby Grapefruit During Cold Storage

**Figure 1:**
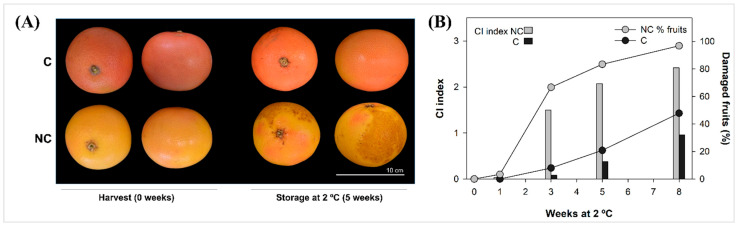
External appearance of covered (C) and non-covered (NC) Star Ruby grapefruit at harvest and after 5 weeks of cold storage (**A**), and chilling injury development shown as CI index (bars) and percentage of damaged fruit (lines) in covered (C) and non-covered (NC) Star Ruby fruits during storage at 2 °C for 8 weeks (**B**).

Bagging of mature-green Star Ruby grapefruits provoked a marked and homogeneous red peel coloration clearly distinguishable from that of sun-exposed fruits, which developed a yellow coloration with pink patches (Figure 1A). This red coloration of covered (C) fruit is determined by accumulation of the red carotene lycopene [7]. Postharvest storage at 2 °C for 8 weeks did not substantially modify peel coloration in either C or non-covered (NC) fruit, in agreement with previous results [6]. However, yellow-pigmented fruits exhibited typical symptoms of CI after the first week of cold storage, which increased progressively, with nearly 100% of the fruits showing severe CI after 8 weeks at 2 °C (Figure 1B).

Contrastingly, red-pigmented C grapefruit displayed a low CI index (CII < 1) throughout the whole storage period, affecting less than 50% of the fruit with slight chilling damage that virtually did not compromise their marketability. These results are similar to that found in previous works and reinforce the tolerance to CI induced by lycopene accumulation in covered (shaded) Star Ruby grapefruits [6,7,13].

### 2.2. Expression of the Genes Involved in Fatty Acid Desaturation in the Flavedo of Covered and Non-Covered Star Ruby Grapefruit During Cold Storage

To analyze whether the differential susceptibility of NC and C Star Ruby grapefruit to develop CI during cold storage may be related to differences in FA desaturation, the relative expression of genes involved in different steps of FA desaturation was evaluated in the flavedo of both fruits at harvest and during 8 weeks of storage at 2 °C.

**Figure 2:**
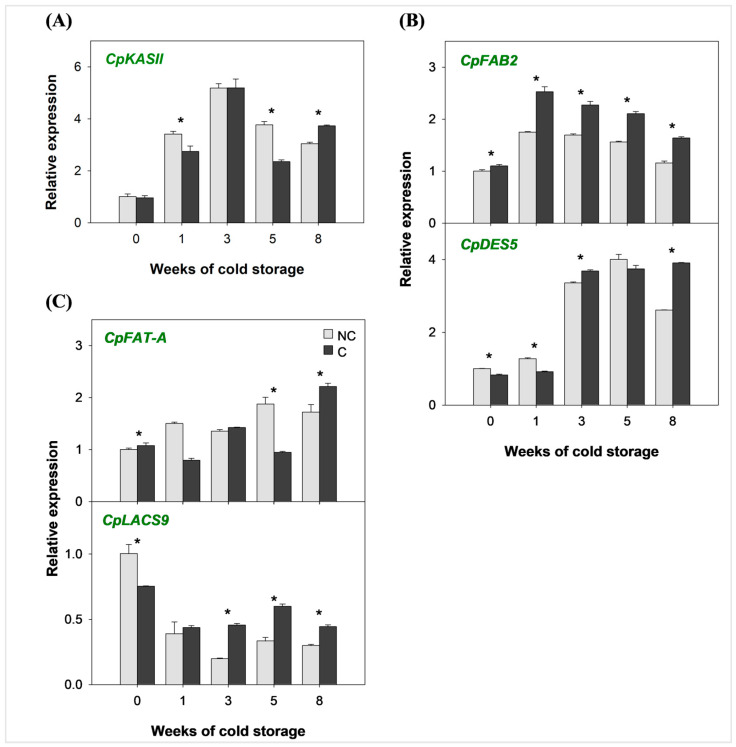
Relative expression of (**A**) β-ketoacyl-ACP synthase II (*CpKASII*); (**B**) stearoyl-ACP desaturases (*SAD*) *CpFAB2* and *CpDES5*; and (**C**) long-chain acyl-CoA synthetase 9 (*CpLACS9*) and fatty acyl-ACP thioesterase A (*CpFAT-A*) gene isoforms in the flavedo of covered (C) and non-covered (NC) Star Ruby grapefruit during 8 weeks of storage at 2 °C. Green gene labels represent enzymatic reactions taking place at the plastid. Data are shown as mean ± SE of three replicates. Asterisks indicate significant difference between NC and C fruit at each sampling date (*p* < 0.05).

The relative expression of *CpKASII*, the gene regulating the elongation step to produce the first C18 fatty acid (Figure 2A), as well as the plastidial isoforms of stearoyl desaturases (*SAD*), *CpFAB2* and *CpDES5*, which are responsible for the desaturation of stearic into oleic acid, increased markedly in response to cold independently of the preharvest shading treatment (Figure 2B). The expression of *CpFAB2* was consistently higher in C than in NC fruits during cold storage, but neither *CpKASII* nor *CpDES5* showed regular differences in both types of fruits. The expression of the plastidial gene *CpFAT-A*, involved in FA modifications for intracellular transport and activation, fluctuated during cold storage, being upregulated in both C and NC fruits after 8 weeks, likely in response to low temperature (Figure 2C). On the other hand, the expression of *CpLACS9* gene, which regulates oleic acid transport from plastids to endoplasmic reticulum, was significantly higher in NC than in C fruits at harvest. However, cold storage downregulated *CpLACS9* expression, and after the third week, this difference was reversed, showing higher levels in C than in NC grapefruits (Figure 2C).

**Figure 3:**
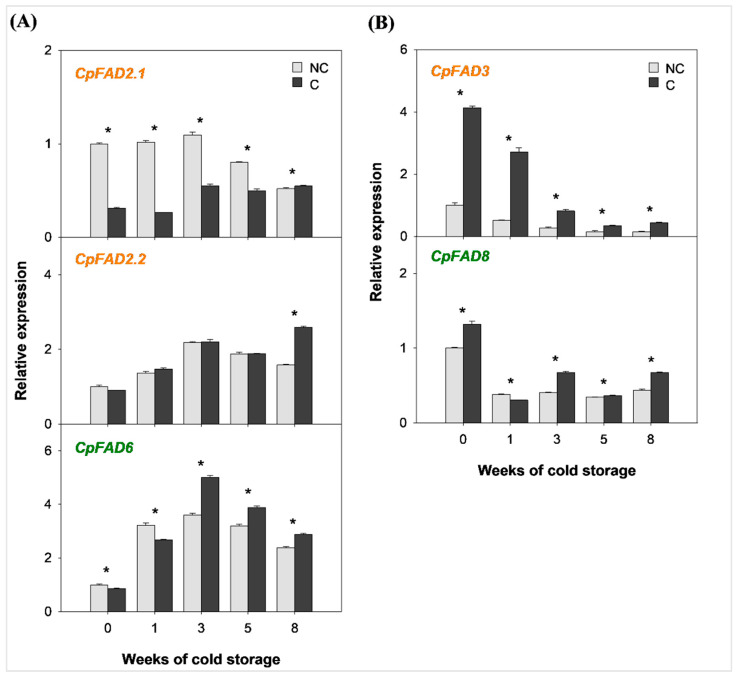
Relative expression of (**A**) endoplasmic (*CpFAD2.1* and *CpFAD2.2*) and plastidial (*CpFAD6*) oleate desaturase gene isoforms and of (**B**) endoplasmic (*CpFAD3*) and plastidial (*CpFAD8*) linoleate desaturase gene isoforms in the flavedo of covered (C) and non-covered (NC) Star Ruby grapefruit during 8 weeks of storage at 2 °C. Green and orange gene labels represent enzymatic reactions taking place at the plastid and the endoplasmic reticulum, respectively. The data are shown as the mean ± SE of three replicates. Asterisks indicate a significant difference between NC and C fruits at each sampling date (*p* < 0.05).

The three isoforms of oleate desaturase genes, regulating the desaturation of oleic to linoleic acid, showed differences in their expression patterns between C and NC grapefruits at harvest and during cold storage (Figure 3A). Accumulation of the endoplasmic *CpFAD2.1* mRNAs was more than three times higher in NC than in C fruits at harvest, and although transcript levels declined slightly in response to cold, they remained significantly higher in NC grapefruits for up to 5 weeks of refrigeration. On the contrary, the endoplasmic isoform *CpFAD2.2* was upregulated during cold storage and showed no differences between both types of fruits. The expression of the plastidial isoform *CpFAD6* displayed the most significant transcriptional upregulation by cold, with a 3-fold increase after 3 weeks at 2 °C in both fruits, and declined thereafter (Figure 3A). The two isoforms of the linoleate desaturase genes, the endoplasmic *CpFAD3* and the plastidial *CpFAD8*, were more highly expressed in C than in NC fruits at harvest (x4 and x1.3, respectively), indicating upregulation by fruit shading (Figure 3B). Interestingly, the expression of both isoforms decreased in both fruits during cold storage, and in both cases, transcript levels in C fruits remained higher than in NC at the end of storage (Figure 3B).

### 2.3. Fatty Acid Content and Composition in the Flavedo of Covered and Non-Covered Star Ruby Grapefruit During Cold Storage

The fatty acid profile in C and NC grapefruits was analyzed by gas chromatography–mass spectrometry (GC-MS) at harvest and throughout cold storage (Table 1). The fatty acid profile in the flavedo of grapefruit at harvest was characterized by linoleic acid as the major FA, accounting for 44–48% of the total content in C and NC fruits, respectively, followed by linolenic (26–24%) and palmitic acids (21–19%) and small proportions of oleic (8–6%) and stearic acids (1–2%).

Quantitative analysis of total FA in the flavedo of fruit at harvest showed a 26.6% lower content in C than in NC fruits, mainly determined by a 30% reduction in linoleic acid and a 15% reduction in palmitic acid as the main significant differences. Consequently, under shaded conditions, the contents of UFA and SFA were lower than in light-exposed fruits, but without a significant effect in their ratio (Table 1). During cold storage, the total FA levels increased in both C and NC fruits, similarly to the UFA and SFA contents. Likewise, with the exception of oleic and stearic acids, the content of individual FAs increased during cold storage independently of the preharvest treatment, but palmitic, linoleic, and linolenic acids were significantly higher in NC than in C grapefruits at the end of the cold storage period. It is noteworthy that the double-bond index (DBI) remained similar between C and NC fruits during the whole pre- and postharvest periods (Table 1).

### 2.4. Multivariate Analysis of Fatty Acid Content and Fatty Acid Desaturase Gene Expression in the Flavedo During Cold Storage of Covered and Non-Covered Star Ruby Grapefruit

Based on the results of the different molecular and biochemical analyses, a principal component analysis (PCA) was carried out to elucidate potential relationships between FA content, the unsaturation level, and the expression of the FA desaturase genes in response to cold storage in the flavedo of covered and non-covered grapefruit.

**Figure 4:**
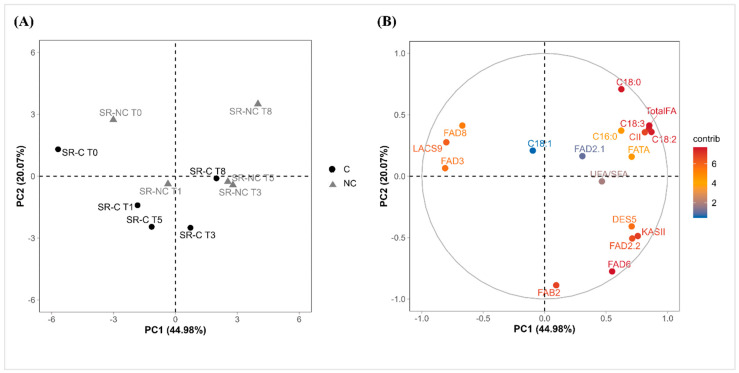
Principal component analysis (PCA) score plot (**A**) and variable plot (**B**) for PC1 vs. PC2 regarding the variables analyzed (CI index, fatty acid content, and relative gene expression) in covered (C) and non-covered (NC) Star Ruby grapefruit. Variables are colored in a gradient from blue (low) to red (high) according to the percentage contribution of each variable in the selected PC axes.

The first two principal components of the analysis explain more than 65% of the total variance of the data (PC1 = 44.98% and PC2 = 20.07%), as represented in the score plot and the variable plot (Figure 4). The samples displayed a common distribution pattern along PC1 in both C and NC groups, mainly discriminated by storage time, whereas PC2 separated the samples according to the bagging treatment (Figure 4A). The variable plot (Figure 4B) revealed that the expression of *CpLACS9*, *CpFAD3*, and *CpFAD8* genes were the variables that contributed most to the separation of C and NC samples at harvest. As represented in the variable plot (Figure 4B), the total FA and linoleic acid (C18:2) contents were major variables contributing to explaining the variance of the data. These two variables presented significant differences between C and NC samples from harvest to 8 weeks of storage (Table 1). Furthermore, the perceptible common pattern in both C and NC samples discriminating by the storage time could be attributed to the results of gene expression (Figure 2, Figure 3 and Figure 4) and FA content (Table 1) in which C and NC fruits followed a similar pattern, although the differences observed at harvest were progressively reduced during the cold storage period.

## 3. Discussion

Changes in fatty acid (FA) metabolism, specifically modification of FA desaturation, have been long associated with the adaptation of sensitive plant species to cold stress as they act as modulators of membrane fluidity to maintain its functionality, conferring tolerance to low temperature [17]. Desaturation of these metabolites is mediated by fatty acid desaturases (FADs), and their transcriptional changes have been studied in the responses of fruit to postharvest cold storage [25,26,30,31,32,33,34,36]. Fruits of different *Citrus* species and varieties exhibited differential susceptibility to the development of chilling injury (CI) symptoms under prolonged storage at cold temperature [2]. The biochemical and molecular basis of this diversity is not well understood but it is generally accepted in the different citrus-producing countries that grapefruit and lemons, both yellow-pigmented fruits, are among the most susceptible *Citrus* species to CI during postharvest cold storage [1].

To assess the involvement of FA metabolism in the tolerance of *Citrus* fruit to CI, the present study evaluated the expression of FA desaturase genes and the fatty acid profile in the flavedo of preharvest covered (C) and non-covered (NC) grapefruits with contrasting CI susceptibility during subsequent cold storage. In previous work, we reported that covering immature-green (around August) Star Ruby grapefruit induced the accumulation of the red carotene lycopene in the peel of mature fruits that were highly tolerant to chilling damage during storage at 2 °C [7]. Contrastingly, sunlight-exposed fruits were almost devoid of carotenoids (naturally yellow-pigmented with pink patches; Figure 1A) and developed CI symptoms during cold storage [4,7] (Figure 1B). Lycopene accumulation in the peel of bagged fruits was also associated with high singlet-oxygen antioxidant capacity (SOAC), over-expression of the antioxidant enzymes CAT and SOD, lower lipid peroxidation [13], and lower tocopherol content [6], rendering CI tolerance during storage at 2 °C. Altogether, shading-induced tolerance to CI in Star Ruby grapefruit offers an excellent system to investigate the involvement of FA desaturation in CI in grapefruits, which are otherwise very prone to developing this postharvest disorder [5].

Fatty acid profiling, determined by GC-MS, in the flavedo of grapefruit at harvest revealed that linoleic (18:2) and linolenic (18:3) acids were the major FAs in this cultivar, followed by palmitic (16:0), oleic (18:1), and stearic (18:0) acids (Table 1), consistent with previous report in fruits of the same variety at commercial maturity growing under different conditions [5]. In comparison with the FA profile described for fruits of other *Citrus* genotypes, it seems that linoleic acid prevailed as the main FA in most of them [26,45,46]. However, regarding the linolenic acid content in grapefruit, there were substantial differences since in fruits of lemon [45], blood oranges [46], and mandarin [26], the proportion of this compound is lower than that of palmitic acid. Thus, the total FA content and individual FA relative proportion appear to be species-dependent and may be modulated by growing conditions [47].

Fruit shading mostly reduced the content of palmitic and linoleic acid at harvest, which resulted in a significant reduction in total FA (24%), unsaturated FA (25%), and saturated FA (20%) over light-exposed fruits. These changes did not affect the UFA/SFA ratio or the double-bond index (Table 1). In fruits of other species, such as olive drupes [30,39], cucumber [40], and walnuts [41], a reduction in UFA has been previously attributed to shading treatment. Although a direct effect of light incidence in the flavedo of C grapefruits cannot be discarded, larger increases in palmitic and linoleic acids were also detected in fruits of the CI-sensitive mandarins than in the CI-tolerant ones at harvest [26]. Studies in other fruit also correlated higher UFA levels with CI-sensitive genotypes [20,48,49], suggesting an attempt of sensitive tissues (as NC grapefruits) to cope with cold stress under field conditions. It is worth mentioning that the natural maturation and harvesting period of grapefruit in the Mediterranean region occurs during the winter season, when fruits are exposed to low temperatures in the field. Under these conditions, it is likely that CI-sensitive fruit activates cold adaptation mechanisms, such as the increase in FA desaturation. Nevertheless, chilling stress is also known to activate the FA oxidation pathway [50], with UFAs serving as preferred LOX substrates [51], together with other stress-related processes. In fact, previous work showed higher MDA levels in NC than in C fruit at harvest, indicating enhanced lipid peroxidation already in the field [13]. Lipid peroxidation may be mitigated in C fruit by lycopene accumulation [7] without requiring the activation of FA desaturation to maintain membrane fluidity. Altogether, this may help to explain the apparent contradiction that CI-sensitive fruits develop CI despite exhibiting higher levels of FA desaturation, likely due to the stimulation of the responses to cold stress before harvest.

During cold storage, total FA and UFA increased in both C and NC fruits (Table 1). Interestingly, although these two parameters mostly contributed to the differences between C and NC samples over cold storage (Figure 4), the relative increment after 8 weeks of storage at 2 °C in these parameters was very similar for both types of fruits (48% increase in both total FA and UFA; Table 1). These results clearly suggest that the stimulation of FA metabolism towards increasing the desaturation level in postharvest cold storage is a common response of Star Ruby grapefruits regardless of their sensitivity/tolerance to CI development. This observation is consistent with previous findings in other *Citrus* genotypes [26,46,52,53]. Therefore, modulating the FA content and composition during postharvest cold storage appears to be a common response of C and NC grapefruits, while the UFA increase in CI-sensitive (NC) grapefruits appears to be an adaptive mechanism previously activated under preharvest conditions, similarly to that observed in the CI-sensitive Fortune mandarin [26].

The expression of 10 genes related to FA desaturation in the peel of NC and C Star Ruby grapefruit revealed striking differences at harvest and after cold storage. Genes involved in the early stages of FA synthesis and desaturation in the plastid (Figure 2) exhibited a general upregulation during cold storage in both C and NC fruits. This effect was particularly pronounced for *CpKASII*, which showed a 3-fold increase as early as one week after storage (Figure 2A) in both NC and C fruits. The upregulation of this elongase gene in grapefruit is similar to that observed in fruits of the cold-sensitive Fortune mandarin [26] and appears to be independent of light exposure. The expression of plastidial stearoyl-ACP desaturases genes (Figure 2B), involved in the conversion of stearic to oleic acid, showed earlier (*CpFAB2*) or later increase (*CpDES5*) during cold storage depending on the isoform. These marked changes could suggest a stress-related response in cold-sensitive genotypes not influenced by light avoidance under field conditions. However, it is noteworthy that despite the upregulation of genes of this pathway, the levels of stearic and oleic acid did not significantly change during cold exposure (Table 1), suggesting that they may be used as substrate for further desaturations during cold storage or other post-transcriptional mechanisms affecting the activities of the enzymes. Moreover, the genes involved in inter-organelle oleic acid transport displayed contrasting expression patterns in response to cold exposure, with a progressive increase in *CpFAT-A* but a reduction in *CpLACS9* transcripts (Figure 2C). This behavior is similar to that shown for cold-stored mandarin fruits [26], indicating a specialized function for each enzyme activity in fatty acid transport. Interestingly, in cold-sensitive fruits, either NC Star Ruby or Fortune mandarin [26], *CpLACS9* was more highly expressed at harvest than in tolerant fruits, suggesting that this step may be responsive to changes in field conditions in CI-sensitive genotypes. The implication of *LACS9* in cold adaptation has been recently noted in other horticultural species, such as melon [54] and pepper [55]. Therefore, preharvest upregulation of this gene could be an attempt to achieve cold adaptation in the field of CI-sensitive varieties that may later fail during lower storage temperatures.

The expression of *CpFAD2.1*, an endoplasmic isoform of oleate desaturase genes, showed one of the most striking differences between NC and C grapefruits at harvest, which was maintained until 5 weeks of storage at 2 °C (Figure 3A). The downregulation of *FAD2* by shading has been reported in other species, such as walnut embryos [41] and cotton [43], and by darkness in olive drupes [42]. However, in mandarin fruit exposed to regular light conditions in the field, *CpFAD2.1* was more highly expressed in the flavedo of CI-sensitive genotypes, indicating a protective preharvest response of sensitive mandarins maintained during cold storage, which does not take place in resistant varieties [26]. In Star Ruby grapefruits, the differences observed between NC and C fruits at harvest may be largely related to the absence of light in C fruits, although the role of the tolerance to CI induced by lycopene accumulation cannot be discarded. Thus, the greatest accumulation of *CpFAD2.1* transcripts in the CI-sensitive NC grapefruits would reinforce the observations in mandarin fruits and the motion of a potential preharvest response to cold under field conditions [26]. The other isoforms of oleate desaturase, the endoplasmic *CpFAD2.2* and the plastidial *CpFAD6*, displayed similar expression patterns in both C and NC fruits, increasing at the beginning of cold storage (Figure 3A), indicative of a conserved cold response independent of preharvest light incidence. It is worth mentioning that the content of polyunsaturated linoleic acid, the immediate product of oleate desaturase activity, was higher in the flavedo of NC than C fruits, and its levels could probably be attributed to the activity of the endoplasmic isoform *CpFAD2.1*, which markedly increased in NC fruits at harvest (Table 1).

Finally, the two isoforms of linoleate desaturases, *CpFAD3* and *CpFAD8*, involved in the synthesis of the unsaturated linolenic acid, were more highly expressed in the CI-tolerant than in CI-sensitive grapefruits at harvest, especially the endoplasmic *CpFAD3* isoform (Figure 3B). The regulation of these genes by light in other plants has been shown to be species-dependent, since *CpFAD8* was upregulated under dark conditions in oil palm [56], whereas the expression of *CpFAD3* and *CpFAD8* was downregulated under darkness and recovered after light exposure in soybean cell suspensions [44]. In grapefruit, accumulation of these mRNAs seems to be light-sensitive as their expression was clearly enhanced in C fruits (Figure 3B). However, the expression of *CpFAD3* and *CpFAD8* also appears to be temperature-sensitive as it sharply decreased as a cold response from the beginning of storage in C fruits and remained downregulated in NC fruits (Figure 3B). Different examples of the influence of temperature in the expression of linoleate desaturases have been reported in other plant species [57,58,59]. Furthermore, light and temperature have been found to influence not only transcription but also post-transcriptional [44] and post-translational [60,61] stability of linoleate desaturases, what could explain why linolenic acid levels are not higher in C fruit at harvest despite the greater expression of these enzymes. The downregulation of these genes in coordination with the increasing expression of the oleate desaturase *CpFAD2.2* and *CpFAD6* during cold conservation may contribute to the increase in linoleic acid (18:2) observed in both NC and C fruits as a common cold response during storage at 2 °C (Table 1). However, the increase in linolenic acid (18:3) over cold storage in both treatments (Table 1) may be influenced by different factors other than the transcription of these enzymes, as the accumulation of a specific FA is the result of a complex metabolic balance.

To summarize, transcriptional changes in most *FAD* genes were detected from the first week of cold storage, with similar expression patterns across isoforms in both C and NC grapefruits. This suggests a conserved response to cold storage in grapefruits, regardless of their susceptibility to CI, similar to that observed in mandarins [26]. However, significant differences between C and NC fruits were already noticeable at harvest, particularly the transcriptional upregulation of oleate desaturase isoforms in CI-sensitive fruit and of linoleate desaturase isoforms in shaded fruit, indicating that preharvest factors, likely field temperature and light, may be implicated in their regulation. Furthermore, linoleic acid levels, as well as total FAs and UFAs, were clearly stimulated before harvest in the flavedo of NC compared to C grapefruits, consistent with observations in CI-sensitive and CI-tolerant mandarins [26]. These results support the idea that a potential cold adaptation response in CI-sensitive fruit may already be initiated in the field. Altogether, our findings highlight the strong influence of preharvest factors on postharvest CI susceptibility in *Citrus* fruits that may help to develop management strategies under field conditions to prevent or reduce CI during postharvest storage.

## 4. Materials and Methods

### 4.1. Plant Material and Storage Conditions

Fruit of Star Ruby grapefruit (*Citrus paradisi*) from a commercial orchard located in Llíria (València, Spain) were used. Immature-green fruit located outside the canopy was covered with black polyethylene plastic bags around the end of July, as previously described [4]. Covered (C) and non-covered (NC) fruit, exposed to natural environmental conditions, were harvested at full maturity (December) and selected by color uniformity as described in [6]. Fruits were randomly separated into two lots and stored at 2 °C and 80–85% RH for up to 8 weeks. The first lot, consisting of three replicates of 10 fruits each, was used for non-destructive evaluation of chilling injury (CI), and the second lot, comprising three replicates of 6 fruits each, was used for sampling fruits at 0, 1, 3, 5, and 8 weeks of cold storage. Flavedo tissue was excised with a scalpel and immediately frozen in liquid nitrogen, ground to a fine powder with liquid nitrogen, and stored at −80 °C until analysis.

### 4.2. Chilling Injury Evaluation

At weekly intervals, fruits were inspected for chilling injury symptoms and classified according to the severity and extension of the damage using the following scale: 0, non-damage; 1, low damage (<25% peel surface); 2, moderate damage (25–50% peel surface); and 3, severe damage (>50% peel surface). Chilling injury index (CII) was calculated as described in [6]. The percentage of damaged fruit (with visible CI symptoms independently of the category) was also calculated.

### 4.3. RNA Extraction and cDNA Synthesis

Total RNA was isolated from the flavedo tissue of each replicate (a cluster of ten fruit) at each sampling date using RNeasy Plant Mini Kit (Qiagen, Madrid, Spain). Purification of RNA was performed using DNAse I (DNA free, DNase treatment and removal, Ambion, Barcelona, Spain), and RNA quality was confirmed by 1% agarose gel electrophoresis with Good View^®^ Nucleic Acid Stain (SBS Genetech, Beijing, China). Reverse transcription of 5 µg of total RNA was implemented with SuperScript III Reverse Transcriptase (Invitrogen, Barcelona, Spain) in a total volume of 20 µL. One microliter of first strand cDNA, containing approximately 100 ng of cDNA, was used for each amplification reaction.

### 4.4. Gene Expression Analysis by Quantitative Real Time PCR

The selected genes analyzed in this study codify for enzymes involved in FA desaturation, and the sequences of the designed primers for their amplification are described in the Supplementary Data (Table S1). The relative expression levels of each gene were quantified using quantitative real-time PCR (qRT-PCR) using the LightCycler 480 thermocycler (Roche, Madrid, Spain) and the SYBR Green I Master Kit (Roche, Madrid, Spain). One microliter of first-strand cDNA, containing approximately 100 ng of cDNA, was used for each amplification reaction, and reaction conditions were set followed the manufacturer’s instructions. Briefly, the PCR protocol consisted of pre-incubation at 95 °C for 10 min and 40 cycles of denaturation at 95 °C for 10 s, annealing at 59 °C for 10 s, and extension at 72 °C for 10 s. For gene expression measurements, LightCycler 480 Software release 1.5.0, version 1.5.0.39 (Roche, Madrid, Spain), was utilized. Relative expression calculations were performed using the Relative Expression Software Tool 2002 original version [62], with the expression level of each gene in the flavedo of the ‘Star Ruby non-covered’ sample at harvest serving as the reference sample (assigned an expression value of 1). The housekeeping gene *ACTIN* was used for normalization [6].

### 4.5. Fatty Acids Extraction and Esterification into FAMEs

Fatty acids from the flavedo of grapefruit samples were prepared using a one-step lipid extraction and transesterification based on [63]. In this procedure, 150 mg of lyophilized flavedo sample was dissolved in 3 mL of 2% H_2_SO_4_–methanol, together with 100 µL of methyl heptadecanoate (10 mg mL^−1^) used as an internal standard. The mixture was heated at 80 °C for one hour, enabling single-phase digestion and lipid transmethylation. After cooling at room temperature, 1 mL of hexane and 10 mL of an aqueous solution of NaCl (10%) were added. Two phases were formed, and the upper one containing the fatty acid methyl esters (FAMEs) in hexane was recovered in a vial for GC-MS analysis.

### 4.6. FAMEs Identification and Quantification by GC-MS

The analysis of FAMEs was conducted using a DB-FastFAME capillary column (30 m × 0.25 mm ID × 0.25 μm film thickness) from Agilent Technologies (Santa Clara, CA, USA). The system comprised an Agilent 6890N gas chromatograph coupled with an Agilent 5975B Inert XL MSD (Agilent Technologies, Santa Clara, CA, USA). Sample injections of 1 µL were performed at 250 °C with a split ratio of 5:1. Helium was used as the carrier gas at a flow rate of 1 mL min^−1^. The column temperature program started at 50 °C for 30 s, increased to 194 °C at a rate of 25 °C min^−1^, and then to 245 °C at a rate of 5 °C min^−1^. The positive ion spectrum (mass range 30 to 650 m/z) was recorded using electron impact ionization at an energy of 70 eV. The temperatures of the interface and the ion source were set at 230 °C and 150 °C, respectively. Peak areas for each compound were normalized based on reaction efficiency calculated using the internal standard, methyl heptadecanoate.

Metabolite identification was conducted using the NIST Mass Spectral Library, version 2010, and mass spectral similarities. The identification of fatty acids was further confirmed by comparison with retention times from an external standard mixture of fatty acid methyl esters (Supelco 37 Component FAME mix, Sigma-Aldrich, Saint Louis, MO, USA). Quantification of metabolites was performed using fatty acid-specific calibration curves derived from the same external standard mixture. Concentrations were expressed as µg mg^−1^ of fresh weight. Total fatty acid content was calculated as the sum of unsaturated fatty acids (UFAs; oleic, linoleic, and linolenic acids) and saturated fatty acids (SFAs; palmitic and stearic acids), with the general degree of unsaturation expressed as the ratio of total UFAs to total SFAs (UFA/SFA ratio). The double-bond index (DBI) was calculated by multiplying the percentage of each fatty acid by its number of double bonds and dividing by 100 as in [5]. Results are presented as the mean ± standard deviation of two replicates extracted from each sample.

### 4.7. Experimental Design and Statistical Analysis

A completely randomized experimental design was used. Results of CI evaluation and gene relative expression are presented as mean ± standard errors (SE), whereas results of fatty acid content are expressed as mean ± standard deviation (SD). Differences between the means of covered and control fruit were evaluated with an unpaired t-test, with significance defined at *p* < 0.05. Gene expression data were analyzed using REST 2009 software [62] to assess statistical significance, applying a pairwise fixed reallocation randomization test. Principal component analysis (PCA) was built using data of chilling injury, fatty acid content, and relative gene expression at harvest and during cold storage sampling times for C and NC fruits, implemented in R software, version 4.5.1 [64]. The loading coefficients of the variables analyzed can be found in Table S2.

## Figures and Tables

**Table 1:** Contents (µg·mg^−1^ FW, fresh weight) of total and individual fatty acids and the UFA/SFA ratio and DBI in the flavedo of covered (C) and non-covered (NC) Star Ruby grapefruits stored at 2 °C for 8 weeks. The data are shown as the mean ± SD of three replicates. Asterisks indicate a significant difference between NC and C fruits at each sampling date (*p* < 0.05).

Content (µg·mg^−1^ FW)		Weeks at 2 °C				
		0	1	3	5	8
Palmitic acid (16:0)	NC	1.90 ± 0.05 *	1.71 ± 0.38	2.03 ± 0.12	1.68 ± 0.05	2.83 ± 0.27 *
	C	1.61 ± 0.16 *	2.02 ± 0.04	2.14 ± 0.35	1.80 ± 0.41	2.24 ± 0.08 *
Stearic acid (18:0)	NC	0.23 ± 0.09	0.22 ± 0.01 *	0.22 ± 0.04	0.19 ± 0.01 *	0.35 ± 0.21
	C	0.12 ± 0.10	0.09 ± 0.06 *	0.11 ± 0.09	0.12 ± 0.02 *	0.25 ± 0.05
Oleic acid (18:1)	NC	0.57 ± 0.25	0.57 ± 0.04	0.60 ± 0.11	0.53 ± 0.04 *	0.56 ± 0.11
	C	0.58 ± 0.07	0.59 ± 0.08	0.61 ± 0.07	0.39 ± 0.06 *	0.44 ± 0.05
Linoleic acid (18:2)	NC	4.80 ± 0.18 *	4.54 ± 0.32	5.61 ± 0.29	5.16 ± 0.27	7.06 ± 0.33 *
	C	3.32 ± 0.75 *	4.58 ± 0.06	5.31 ± 1.60	4.03 ± 1.90	5.38 ± 0.01 *
Linolenic acid (18:3)	NC	2.40 ± 0.37	2.49 ± 0.07	3.13 ± 0.15	3.34 ± 0.29	3.95 ± 0.20 *
	C	1.95 ± 0.68	2.73 ± 0.00	2.37 ± 1.18	2.28 ± 1.46	2.83 ± 0.03 *
Total FA	NC	9.91 ± 0.16 *	9.53 ± 0.04	11.60 ± 0.48	10.91 ± 0.47	14.74 ± 1.11 *
	C	7.57 ± 1.56 *	10.00 ± 0.09	10.55 ± 3.10	8.62 ± 3.82	11.15 ± 0.17 *
DBI	NC	1.75 ± 0.09	1.80 ± 0.09	1.83 ± 0.02	1.91 ± 0.04	1.80 ± 0.04
	C	1.71 ± 0.12	1.79 ± 0.01	1.72 ± 0.14	1.73 ± 0.19	1.77 ± 0.03
ΣUFA	NC	7.78 ± 0.30 *	7.60 ± 0.35	9.34 ± 0.56	9.03 ± 0.52	11.56 ± 0.63 *
	C	5.85 ± 1.50	7.90 ± 0.01	8.30 ± 2.84	6.70 ± 3.42	8.65 ± 0.04
ΣSFA	NC	2.13 ± 0.14 *	1.93 ± 0.38	2.26 ± 0.08	1.88 ± 0.06	3.18 ± 0.48 *
	C	1.71 ± 0.06	2.11 ± 0.11	2.25 ± 0.26	1.93 ± 0.40	2.49 ± 0.13
UFA/SFA	NC	3.67 ± 0.39	4.03 ± 0.98	4.14 ± 0.39	4.82 ± 0.43	3.66 ± 0.35
	C	3.38 ± 0.75	3.75 ± 0.20	3.64 ± 0.84	3.37 ± 1.08	3.48 ± 0.17

## Data Availability

The original contributions presented in the study are included in the article/supplementary material, further inquiries can be directed to the corresponding author.

## Supplementary Materials

The following supporting information can be downloaded at https://www.mdpi.com/article/10.3390/plants14243848/s1. Table S1. List of primer sequences used for RT-qPCR analysis. Table S2. Loading coefficients of the variables in the PCA.

## Abbreviations

The following abbreviations are used in this manuscript:

CI	Chilling injury
FA	Fatty acids
C	Covered fruit
NC	Non-covered fruit
FAD	Fatty acid desaturases
UFA	Unsaturated fatty acids
SFA	Saturated fatty acids
CII	Chilling injury index
DBI	Double-bond index
